# An integrated dataset of malaria notifications in the Legal Amazon

**DOI:** 10.1186/s13104-020-05109-y

**Published:** 2020-06-03

**Authors:** Lais Baroni, Marcel Pedroso, Christovam Barcellos, Rebecca Salles, Samella Salles, Balthazar Paixão, Alvaro Chrispino, Gustavo Guedes, Eduardo Ogasawara

**Affiliations:** 1 Federal Center for Technological Education of Rio de Janeiro, CEFET/RJ, Rio de Janeiro, Brazil; 2 Oswaldo Cruz Foundation, Fiocruz, Rio de Janeiro, Brazil; 3 Federal Institute of Rio de Janeiro, IFRJ, Rio de Janeiro, Brazil

**Keywords:** Malaria, Health regions, Legal Amazon

## Abstract

**Objectives:**

Malaria is an infectious disease that annually presents around 200,000 cases in Brazil. The availability of data on malaria is crucial for enabling and supporting studies that can promote actions to prevent it. Therefore, the goal of this paper is to contribute to such studies by offering an integrated dataset containing data on reported and suspected cases of malaria in the Brazilian Legal Amazon comprising the period from the years 2009 to 2019.

**Data description:**

This paper presents a dataset with all medical records of patients who were tested for malaria in the Brazilian Legal Amazon from 2009 to 2019. The dataset has 40 attributes and 22,923,977 records of suspected cases of malaria. Around 12% of the data correspond to confirmed cases of malaria. The attributes include data regarding the notifications, examinations, as well as personal patient information, which are organized into health regions.

## Objective

Since 2003, the Health Surveillance Secretariat of the Ministry of Health implemented, in Brazil, the Malaria Epidemiological Surveillance Information System (SivepMalaria), which is a malaria monitoring system in nine Brazilian states of the Brazilian Legal Amazon (for short, Legal Amazon). The Legal Amazon is the region most susceptible to malaria in the country, comprehending more than 90% of the malaria cases in Brazil [1].

All suspected or confirmed cases of malaria are be notified and registered in SivepMalaria [2]. The information system consists of modules that record data regarding notifications, examinations, as well as personal patient information [3]. All SivepMalaria records are yearly organized and localized according to counties. Thus, SivepMalaria is an important tool for understanding the distribution of malaria and should be used to control the endemy [4]. The data from SivepMalaria are maintained and made available by the Department of Informatics of the Unified Health System of Brazil (DATASUS).

In Brazil, the Unified Health System (SUS) is responsible for providing public health services to the entire population. As a way of organizing these services, the Brazilian territory is divided into health regions. Each health region is organized as a set of counties that must be able to promote health and prevent diseases for the counties it encompasses, including endemic diseases, such as malaria. Analyzing the performance of health regions in care and prevention of malaria is an important matter in the Legal Amazon.

Therefore, the main contribution of this work is to provide an integrated dataset of malaria notifications (for short, IntegratedDataset) [5]. The IntegratedDataset is a fusion of yearly records of SivepMalaria enriched with health regions. Data cleaning and data preprocessing techniques were also applied to improve its quality. All records were translated from Brazilian Portuguese to English to increase the potential use of the integrated dataset.

## Data description

In the area of healthcare, the process of Knowledge Discovery from Databases (KDD) may enable diagnostics, treatments, as well as preventive measures [6–9]. The dataset presented in this paper is targeted precisely for such a goal. It results from a process of data integration organized into three main activities: (i) data fusion, (ii) data enrichment, and (iii) data preprocessing. It is important to emphasize that all criteria adopted for data management were based on detailed studies of the dataset and support from experts in the field.

### Data fusion

Data fusion was applied over data from SivepMalaria yearly collected since 2009, configuring the fusion of all SivepMalaria records (for short SivepMalariaFus). Since SivepMalaria was implemented, its schema has suffered changes throughout the years, including new variables or modifying categories in the same variable. Nevertheless, the integrated dataset developed in this paper provides a unified schema by means of a correspondence table. It contains 40 attributes from the SivepMalaria database containing 22,923,977 records. Among these records, about 12% corresponds to positive cases of malaria.

The selected dataset attributes comprise data of notifications, examinations, and personal patient information. Most of these attributes are categorical and present encoded values. The relationship between the codes and their meanings are translated using a data dictionary.^1^

### Data enrichment

The health regions are part of the systemic organization of the public health of Brazil, aiming at political-administrative decentralization and completeness of assistance. Since the SivepMalariaFus does not include this information, it had to be obtained from another data source. For that, two additional datasets were used for enriching the data contained in the SivepMalariaFus. Respectively, they regard: (i) health regions information (tb_regsaud) and (ii) the relationship between counties and health regions (rl_municip_regsaud). These tables are provided by DATASUS.^2^

The enrichment led to the creation of three new attributes: notification.hr, infection.hr and home.hr. They correspond respectively to the health regions in which the notification and infection occurred as well as to the residence of the infected patient.

### Data preprocessing

After the processes of data fusion and enrichment, data preprocessing was performed. Preprocessing comprehend the application of several techniques for data preparation, that can encompass from the correction or removal of incorrect data to the adjustment of data formatting corresponding to the data mining algorithms used. Among the several preprocessing techniques widely approached in literature, the ones selected for application in our study were (i) attribute selection, (ii) data cleaning, and (iii) data transformation.

### IntegratedDataset

The list of the attributes of IntegratedDataset together with the entire data preprocessing description and its R script is available^3^ [5]. Table 1 provide an overview of all data files/data sets created in this Data note and available for download in the Synapse repository. Additionally, an exploratory analysis using the IntegratedDataset is also available^4^.

**Table 1:** Overview of data files/data sets

Label	Name of data file/data set	File type	Synapse ID
Data set 1	integrated_dataset	delimited text (.csv)	https://www.synapse.org/#!Synapse:syn21555930 [5]
Data set 2	rl_municip_regsaud	delimited text (.csv)	https://www.synapse.org/#!Synapse:syn21552452 [5]
Data set 3	tb_regsaud	delimited text (.csv)	https://www.synapse.org/#!Synapse:syn21552453 [5]
Data file 1	data_integration_process	R code (.r)	https://www.synapse.org/#!Synapse:syn21555551 [5]
Data file 2	dictionary	delimited text (.csv)	https://www.synapse.org/#!Synapse:syn21555933 [5]
Data file 3	dicionario_de_dados_sivep	document (.pdf)	https://www.synapse.org/#!Synapse:syn21555932 [5]
Data file 4	scheme_attribute	delimited text (.csv)	https://www.synapse.org/#!Synapse:syn21568686 [5]
Data file 5	exploratory-analysis	document (.pdf)	https://www.synapse.org/#!Synapse:syn21585579 [5]
Data file 6	integrated-dataset	document (.pdf)	https://www.synapse.org/#!Synapse:syn21585580 [5]

## Limitations

- Personal patient information is only provided for those who tested positive for malaria.
- Some attributes contain more than 80% of missing values. The data dictionary presents the completeness of each attribute in the IntegratedDataset. No data imputation technique has been applied.
- Some values do not add significant information to the research. For example, in the occupation attribute, more than 50% of the fields that are filled correspond to the values “ignored” or “others”.
- To reinforce privacy, we have chosen not to use the attributes of localities (infection and residence) available in the original dataset of SivepMalaria. Localities are smaller than counties and provide very specific information. Inevitably, disregarding this information is a limitation.

## Data Availability

The dataset generated during the current study and additional documentation is freely and openly available on the Synapse repository at https://doi.org/10.7303/syn21552203 [5]. The authors are committed to keeping the IntegratedDataset updated. This means that the IntegratedDataset will be updated whenever new data referring to SivepMalaria are made available by DATASUS, which is expected to be done annually. The new data to be included will undergo the same treatment process described in this paper.

## Abbreviations

SivepMalaria: Malaria Epidemiological Surveillance Information System
Legal Amazon: Brazilian Legal Amazon
DATASUS: Department of Informatics of the Unified Health System of Brazil
SUS: Unified Health System
IntegratedDataset: Integrated Dataset of malaria notifications
KDD: Knowledge Discovery from Databases
SivepMalariaFus: Data fusion over data from SivepMalaria yearly collected since 2009
IBGE: Brazilian Institute of Geography and Statistics

## Footnotes

1 The sivep dictionary can be found at https://is.gd/2ijcpC.

2 These tables can be found at ftp://ftp.datasus.gov.br/territorio/tabelas.

3 https://www.synapse.org/#!Synapse:syn21585580, https://www.synapse.org/#!Synapse:syn21555551.

4 https://www.synapse.org/#!Synapse:syn21585579.
